# CONDUCTING A COUNTYWIDE ALZHEIMER’S DISEASE AND RELATED DEMENTIAS CAREGIVER LANDSCAPE ANALYSIS

**DOI:** 10.1093/geroni/igad104.2833

**Published:** 2023-12-21

**Authors:** D’Artagnan Robinson, Noel Barragan, Monique Parrish, Donna Benton, Mariana Reyes, Susanna Mage, Tony Kuo

**Affiliations:** Los Angeles County Department of Public Health, Los Angeles, California, United States; Los Angeles County Department of Public Health, Los Angeles, California, United States; LifeCourse Strategies, Orinda, California, United States; University of Southern California, Los Angeles, California, United States; Los Angeles County Department of Public Health, Los Angeles, California, United States; University of Southern California, Los Angeles, California, United States; Los Angeles County Department of Public Health, Los Angeles, California, United States

## Abstract

In Los Angeles County (LAC), approximately 1 in 5 caregivers provide care to older adults with dementia and cognitive impairment. By 2040, a projected 1.5 million Californians will be living with Alzheimer’s disease and related dementias, foreshadowing an unprecedented need for family caregivers across the state. In 2021, the LAC Department of Public Health launched the Centers for Disease Control and Prevention-funded LA BOLD initiative with the intent of developing a strategic plan to address dementia awareness and care in LAC, including improving local infrastructure for supporting unpaid family caregivers. To support this effort, LA BOLD commissioned the University of Southern California Family Caregiver Support Center to conduct a landscape analysis of the caregiver population in LAC. The analysis used a multi-method approach that included: primary analysis of community-based organization surveys, stakeholder interviews, focus groups with family caregivers, and secondary analyses of California Health Interview Survey and California Caregiver Resource Center System CareNav™ data and an environmental scan of best-practice caregiving models in use in other states. Results informed the development of three functional recommendations specific to LAC caregivers: 1) Leverage existing infrastructure to strengthen and build family caregiver services and supports; 2) Promote health care payer, provider, and system recognition of and support for family caregivers; and 3) Increase respite services for caregivers. This presentation highlights the methods and the findings from the landscape analysis, providing insights into the barriers and opportunities that a growing caregiver population of persons with dementia may pose to LAC.

